# Triple nitrogen-vacancy centre fabrication by C_5_N_4_H_*n*_ ion implantation

**DOI:** 10.1038/s41467-019-10529-x

**Published:** 2019-06-13

**Authors:** Moriyoshi Haruyama, Shinobu Onoda, Taisei Higuchi, Wataru Kada, Atsuya Chiba, Yoshimi Hirano, Tokuyuki Teraji, Ryuji Igarashi, Sora Kawai, Hiroshi Kawarada, Yu Ishii, Ryosuke Fukuda, Takashi Tanii, Junichi Isoya, Takeshi Ohshima, Osamu Hanaizumi

**Affiliations:** 1 Graduate School of Science and Technology, Gunma University, 1-5-1 Tenjin, Kiryu, Gunma, 376-8515 Japan; 2 Takasaki Advanced Radiation Research Institute, National Institutes for Quantum and Radiological Science and Technology, 1233 Watanuki, Takasaki, Gunma, 370-1292 Japan; 3 Institute for Quantum Life Science, National Institutes for Quantum and Radiological Science and Technology, 4-9-1 Anagawa, Inage-ku, Chiba, Chiba 263-8555 Japan; 4 National Institute for Materials Science, 1-1 Namiki, Tsukuba, Ibaraki, 305-0044 Japan; 5 National Institute of Radiological Sciences, National Institutes for Quantum and Radiological Science and Technology, 4-9-1 Anagawa, Inage-ku, Chiba, Chiba, 263-8555 Japan; 6 Faculty of Science and Engineering, Waseda University, 3-4-1 Ohkubo, Shinjuku-ku, Tokyo, 169-8555 Japan; 7 Faculty of Pure and Applied Sciences, University of Tsukuba, 1-1-1 Tennodai, Tsukuba, Ibaraki, 305-8573 Japan

**Keywords:** Condensed-matter physics, Qubits

## Abstract

Quantum information processing requires quantum registers based on coherently interacting quantum bits. The dipolar couplings between nitrogen vacancy (NV) centres with nanometre separation makes them a potential platform for room-temperature quantum registers. The fabrication of quantum registers that consist of NV centre arrays has not advanced beyond NV pairs for several years. Further scaling up of coupled NV centres by using nitrogen implantation through nanoholes has been hampered because the shortening of the separation distance is limited by the nanohole size and ion straggling. Here, we demonstrate the implantation of C_5_N_4_H_*n*_ from an adenine ion source to achieve further scaling. Because the C_5_N_4_H_*n*_ ion may be regarded as an ideal point source, the separation distance is solely determined by straggling. We successfully demonstrate the fabrication of strongly coupled triple NV centres. Our method may be extended to fabricate small quantum registers that can perform quantum information processing at room temperature.

## Abstract (web summary)

There is an extensive literature discussing the potential applications of individual nitrogen vacancy centres but some proposed developments require the use of multiple, coupled defects. Here the authors demonstrate a method to fabricate coupled nitrogen vacancy centre triplets.

## Introduction

A nitrogen vacancy (NV) centre in a diamond is a pair that consists of a substitutional nitrogen and an adjacent vacancy; it has spin *S* = 1 and charge −1. The electron spin of the NV centre shows optical initialization through optical pumping, optical readout through spin-dependent fluorescence, coherent manipulation by microwave pulses, and a long coherence time, all at room-temperature^1^. The coherence time, *T*_2,Hahn_ ~ 2 ms at room-temperature, has been reported both for a grown-in NV and an engineered NV in a ^12^C-enriched diamond film grown by chemical vapour deposition (CVD)^2–4^. These excellent solid-state spin quantum bit (qubit) properties make the NV centre an outstanding platform for room-temperature quantum information processing^5–10^. A possible architecture of a scalable quantum register is a single NV centre, in which the electron spin is coupled to the nuclear spin(s)^11,12^. Quantum error correction has been shown by a hybrid quantum register that consists of an electron spin, as well as one ^14^N and two ^13^C nuclear spins^13,14^. Another possible architecture is an array of NV centres, in which neighbouring electron spins are coupled by dipole–dipole interactions^15^. The dipole coupling strength decreases with an increase in the separation distance, and the detection limit of the dipole coupling strength is determined by the inverse of the coherence time. The NV centres need to simultaneously satisfy both a shorter separation distance and a longer coherence time.

Ion implantation is a useful technique for fabricating NV centres with nanometre separation. The depth of the NV centre is controlled by the ion energy. In 2005, in-plane targeting was achieved via focusing ion beam implantation^16–19^, while a beam diameter of 100 nm was achieved in 2013^16^ in mask-less implantation using a focused ion beam. The collimated ion implantation via nanometre-sized hole gives a much higher positioning resolution^20–29^. For example, Toyli et al. demonstrated the fine grid of NV centres by nitrogen implantation via a nanohole in 2010^23^. In contrast to ion implantation, Chen et al. proposed a novel technique of fabricating a fine grid of NV centres by the femtosecond laser^30,31^. The advantage of the femtosecond laser is deterministic creation. However, there is still room to improve the separation distance and coherence time owing to fabricate coherently coupled NV centres by this technique. The implantation via a nanohole on a poly (methyl methacrylate) (PMMA) resist mask fabricated by electron beam lithography is commonly utilized for accurately positioning NV centres as of today^24–29^. The in-plane accuracy is determined by the nanohole diameter as well as by straggling. Because the straggling decreases with a decrease in the ion energy, lower energy is effective for accurate targeting. The disadvantage of low energy ion implantation is a short coherence time. While the coherence property of NV centres is disturbed by the surrounding spin bath, the shallow NV centres created by low energy implantation are mostly influenced by surface spins, resulting in a shorter coherence time. In addition to the coherence time, the disadvantage of the low energy is a low creation yield, which is the number of created NV centres divided by the number of implanted nitrogen ions. The vacancies provided by a low energy ion are insufficient for the efficient fabrication of NV centres. From the point of view of coupled multiple NV centres fabrication, the optimum implantation condition considering the ion energy, targeting accuracy, coherence time, and creation yield need to be explored. Recently, Jakobi et al. investigated the implantation conditions in order to fabricate coherently coupled NV pairs at a reasonable probability^25^. An optimal energy of 30 keV was applied for ^14^N^+^ implantation through nanoholes with 50 nm diameters on PMMA. Here, the NV_A_–NV_B_ pairs, for which the dipole–dipole interaction, *ν*_dip_, is measured by double electron–electron resonance (DEER), are denoted coupled pairs, one with strong coupling, *ν*_dip_ > 1/minimum(1/*T*_2A_, 1/*T*_2B_) and one with weak coupling, *ν*_dip_ < 1/minimum(1/*T*_2A_, 1/*T*_2B_), respectively. Six strongly coupled ^14^NV–^14^NV pairs and four weakly coupled ^14^NV–^14^NV pairs were found among the 6,000 implantation sites. Extending the same method to fabricate the coupled triple NV centres is not feasible because searching for an order of 10,000 implantation sites seems to be required^25^. Scarabelli et al. succeeded in creating small nanoholes (8–20 nm) on a thin gold film under PMMA by the combination of the metal etching technique and electron beam lithography^26^. The distance between the NV centres was controlled by the pitch (40 or 60 nm) of the nanohole array. The lower energy of ^15^N^+^-10 keV and the small nanoholes with nanometre gaps contributed to the fabrication of multiple NV centres. However, the separation distance of 40 nm between the NV centres was still too large for coupling. To scale up via the nanohole implantation technique, it is still necessary to achieve shorter distance NV centres and improve the creation yield without decreasing the coherence.

Another approach for creating coupled NV is nitrogen molecular (N_2_) ion implantations^32,33^. When an N_2_ ion hits the diamond it decomposes into two individual nitrogen atoms. Because the targeting accuracy is solely determined by straggling, the molecular ion implantation is regarded as an ideal point source. Yamamoto et al. reported the fabrication of an NV–NV pair with a dipole coupling strength of 55 kHz via ^15^N_2_^+^-20 keV implantation^33^. The NV pair was coupled with a dark spin. The dipole coupling strengths between the dark spin and each NV centre were 172 and 330 kHz. This provided a system of coupled three electron spins. However, a quantum register consisting of three or more NV centres has, to date, never been realized. Gaebel et al. reported NV–N pair fabrication via ^14^N_2_^+^-14 keV implantation^32^. They reported that the estimated separation distance and the dipole coupling strength between ^14^NV and ^14^N were 1.5 nm and 14 MHz, respectively. It is obvious that ion implantation with a higher number of nitrogen atoms in a single molecule, such as N_3_ or N_4_, could lead to further scaling. However, there has been no report of coupled NV centres produced by nitrogen cluster ions, as the formation of a pure nitrogen cluster has been hardly implemented.

In the present work, a ground-breaking solution for scaling-up is innovated by implanting a C_5_N_4_H_*n*_ ion from an adenine (C_5_N_5_H_5_) source. In the same manner as the nitrogen molecular ion, the C_5_N_4_H_*n*_ ion contains multiple nitrogen atoms. The size of a single adenine molecule is estimated to be less than 0.5 nm from its molecular structure^34^. Therefore, the C_5_N_4_H_*n*_ acts as an ideal point source. The improvement in the creation yield was expected from the co-implantation of carbon and hydrogen atoms, which provide vacancies. It is well known that the co-implantation of nitrogen and carbon ions improves the creation yield^27,33,35^. Here, vacancies supplied from carbon ions originating from the same C_5_N_4_H_*n*_ are expected to contribute effectively to increasing the conversion yield of nitrogen from the same ideal point source. A similar increase in the yield by supplying vacancies around the implanted nitrogen was reported for CN^−^ molecular ion implantation^27^. We propose that these features overcome the barrier of scaling-up. The ionization of adenine has already been experimentally examined^36,37^, where the ionized C_5_N_4_H_*n*_ were electrically extracted and implanted into a diamond. When a C_5_N_4_H_*n*_-65 keV ion hits the diamond, it decomposes into individual atoms with energies of 7.7, 6.6, and 0.6 keV for nitrogen, carbon, and hydrogen, respectively. At each implantation spot, four nitrogen atoms (7.7 keV), five carbon atoms (6.6 keV), and about five hydrogen atoms (0.6 keV) are therefore provided from an ideal point source. The spatial distribution of the stopping positions of the implanted atoms within each implanted spot are determined by straggling. In this study, we achieve the fabrication of triple NV centres coherently coupled with a dipole–dipole interaction by using a nitrogen compound ion beam from adenine powders.

## Results

### Creation of multiple NV centres in an optical spot

**Fig. 1:**
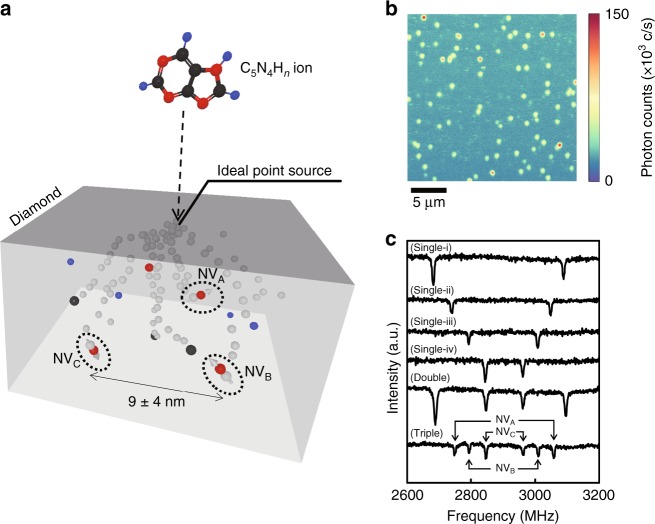
CFM image and ODMR spectra of NV centres fabricated by C_5_N_4_H_*n*_ ion implantation. **a** Fabrication of triple NV centre with nanometre separation by C_5_N_4_H_*n*_ ion implantation. When a C_5_N_4_H_*n*_-65 keV ion hits the diamond, it decomposes into individual atoms. The red, black, and blue spheres represent nitrogen atoms, carbon atoms, hydrogen atoms, respectively. The vacancies are created by these ions and presented by grey spheres. During the annealing process, the vacancies mobilize and combined with substitutional nitrogen, interstitial carbon, and other vacancies. If the vacancy meets substitutional nitrogen, the NV centre is created and labelled by NV_A_, NV_B_, and NV_C_ with dashed circles in the figure. The C_5_N_4_H_*n*_ ion acts as an ideal point source, and the position of each atom is determined by ion straggling. The average distance and standard deviation of the implanted nitrogen atom were calculated to be 9 and 4 nm, respectively, by using the SRIM code. **b** Typical CFM image of C_5_N_4_H_*n*_ ion implanted region. Yellow dots correspond to single NV centres, and bright red dots correspond to double and triple NV centres. **c** Typical ODMR spectra of single, double, and triple NV centres. Top four spectra (Single-i), (Single-ii), (Single-iii), and (Single-iv) show four single NV centres with different orientations. The spectra (Double) and (Triple) show double and triple NV centres, respectively. The spectra for triple NV centre are labelled NV_A_, NV_B,_ and NV_C_

Figure 1a shows a scheme of the triple NV centre created by the implantation of C_5_N_4_H_*n*_-65 keV. When a C_5_N_4_H_*n*_-65 keV ion hits the diamond, it decomposes into individual atoms. The red, black, and blue spheres represent nitrogen atoms, carbon atoms, hydrogen atoms, respectively. The vacancies are created by these ions and presented by grey spheres. During the annealing process, the vacancies diffuse and combined with substitutional nitrogen, interstitial carbon, and other vacancies. If the vacancy meets substitutional nitrogen, an NV centre is created, which are labelled as NV_A_, NV_B_, and NV_C_ with dashed circles in the figure. The C_5_N_4_H_*n*_ acted as an ideal point source, and the final positions of the implanted atoms were determined by ion straggling. The projected ranges and straggling for all atoms were calculated with Stopping and Range of Ions in Matter (SRIM) version 2008^38^. The average distance and standard deviation for nitrogen atoms were calculated to be 9 and 4 nm, respectively. Figure 1b shows a typical image observed with a laboratory-built confocal laser scanning fluorescence microscopy (CFM) system. Isolated spots can be clearly observed in the implanted region with a fluence of ~10^8^ cm^−2^. Photoluminescence spectroscopy and optically detected magnetic resonance (ODMR) identified the origin of the spots as a negatively charged NV centre. Dense NV centres were created when the fluence was ~10^10^ cm^−2^ (Supplementary Fig. 1), and no NV centres were observed in non-implanted regions. The number of NV centres in a fluorescence spot was estimated by the following method. First, the single NV centres were distinct from multiple NV centres based on the photon counting rates associated with different numbers of NV centres^24^. The second-order autocorrelation measurements were performed to confirm that the fluorescence spots contained a single NV centre. We selected the fluorescence spots satisfying *g*^2^(0) < 0.5, where *g*^2^(*τ*) is the second-order autocorrelation function. The typical photon counts from a single NV were 5 × 10^4^ c s^−1^. Second, the number of NV centres in a spot was verified from its ODMR spectrum. Figure 1c shows typical ODMR spectra from single, double, and triple NV centres at a magnetic field of ~9 mT. We called an optical spot consisting of two or three NV centres, as evidenced by an ODMR spectrum arising from differently oriented NV centres, as double or triple, respectively. NV centres with different orientations can be distinguished by their Zeeman splittings. The top four spectra (Single-i), (Single-ii), (Single-iii), and (Single-iv) show four single NV centres aligned with different orientations (labelled axis-i, -ii, -iii, and -iv, respectively). The spectra (Double) and (Triple) show double and triple NV centres, respectively. As shown, three NV centres in the triple NV centre were labelled as NV_A_, NV_B_, and NV_C_, respectively. The three NV centres with different orientations (axis-ii, -iii, and -iv) can be individually addressed by different resonance frequencies, even under homogeneous magnetic fields. The resonant frequency of multiple NV centres can be divided by using local magnetic field gradients, even if they have same orientation^39,40^. The individual addressing is an important feature for coupled multiple NV centres for use as quantum registers. The use of local magnetic field gradients is required in the case of scaling the quantum register to more than five qubits.

### Creation yield and coherence time

**Fig. 2:**
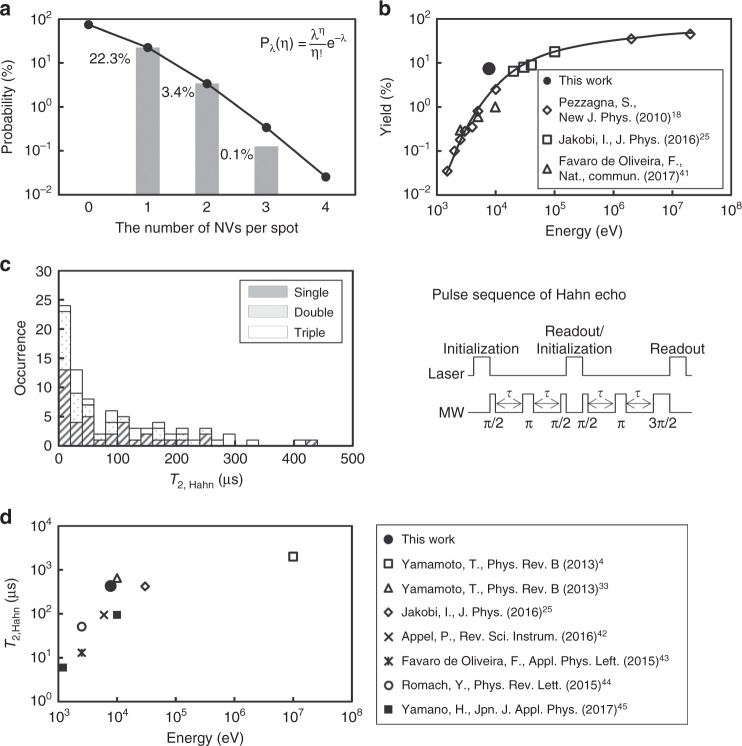
Creation yields and coherence times. **a** Ratio of created fluorescent spots to implanted C_5_N_4_H_*n*_ ions from a total measured area of 10,400 μm^2^. The numbers of single, double, and triple NV centres were 1,589 (22.3%), 244 (3.4%), and 9 (0.1%), respectively. The closed circles and solid line represent the best fitting Poisson distribution (*λ* = 0.30). The fitting was conducted in a linear scale and the plotting is represented in a logarithmic scale. **b** Creation yield depends on the ion acceleration energy. The closed circle shows the creation yield of the NV centres in this study, and the open symbols show the data from previous studies. It is clear that the creation yield of 7.4% achieved in this study is higher than those reported from previous studies. **c** Histogram of the coherence time, *T*_2,Hahn_, measured by Hahn echo pulse sequence shown next to the histogram. The hatched, dotted, and open bars show the *T*_2,Hahn_ values observed from the single, double, and triple NV centres, respectively. The total numbers of measured *T*_2_,_Hahn_ for single, double, and triple NV centres were 38, 13 (26 NVs), and 9 (19 NVs). The maximum *T*_2,Hahn_ in this work was 428 µs. **d** Comparisons of maximum *T*_2,Hahn_ in this work with those from the previous studies with various ion energies

Figure 2a shows the ratio of the created fluorescent spots to the implanted C_5_N_4_H_*n*_ ions for the total measured area of 10,400 μm^2^.  The closed circles and solid line represent the best fitting Poisson distribution. The detail of Poisson fitting is shown in Supplementary Fig. 2. The number of implanted C_5_N_4_H_*n*_ ions was estimated to be 7,116 from the best fitting Poisson distribution by minimizing the residual sum of squares (RSS). As shown in the figure, among a total of 7,116 implantation spots for C_5_N_4_H_*n*_ ions, the numbers of single, double, and triple NV centres were 1,589 (22.3%), 244 (3.4%), and 9 (0.1%), respectively. Next, we evaluated the creation yield of the NV centres, *P*, which is defined as *P* = *N*_NV_/(*n*_N_*N*_ion_) × 100 (%), where *N*_NV_ and *N*_ion_ are the number of NV centres and implanted ions, respectively, and *n*_N_ is the number of nitrogen atoms in an ion. In this study, four nitrogen atoms were present, i.e., C_5_N_4_H_*n*_. The number of implanted ions, *N*_ion_, was estimated to be 7,116. The total creation yield of NV centres was calculated to be 7.4%. Figure 2b shows the creation yield as a function of the ion energy^18,25,41^. The total creation yield in this study was higher than the previously reported data for nitrogen ion implantation, such as 1% for ^14^N-9.8 keV ions and 2.5% for ^14^N-10 keV ions^18,41^.

Figure 2c shows a histogram of the coherence time, *T*_2,Hahn_, measured using a Hahn echo pulse sequence. The coherence of the NV centre was investigated with two consecutive near-identical pulse sequences, as shown in the figure. The measured *T*_2,Hahn_ are listed in Supplementary Table 1. The hatched, dotted, and open bars show the *T*_2,Hahn_ values for single, double, and triple NV centres, respectively. The longest achieved coherence time was 428 μs. Figure 2d shows the coherence time^4,25,33,42–45^ as a function of the ion energy. The maximum values in each paper are represented. The coherence time increased with an increasing ion energy. The coherence time strongly depends on the surface conditions, crystal quality, and annealing conditions. In this study, a reasonable coherence time was given compared to the previously reported data.

### Double electron–electron resonance (DEER)

**Fig. 3:**
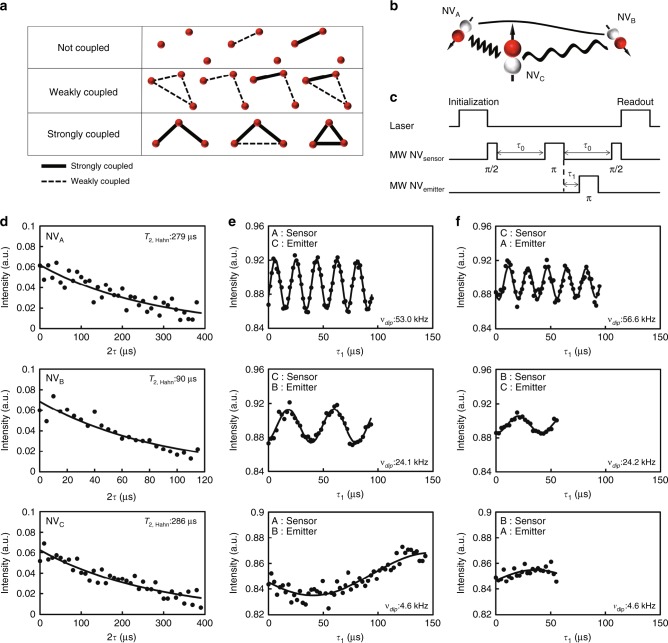
Coherence times and DEER of triple NV centre. **a** Classification of triple NV centres. **b** Schematic diagram of evaluated triple NV centres. **c** Pulse sequence in DEER measurements. **d** Hahn echo decay curves for NV_A_, NV_B_, and NV_C_. The closed circles represent the experimental echo data and the solid lines show the exponential fitting curves. The *T*_2,Hahn_ values for NV_A,_ NV_B_, and NV_C_ were evaluated to be 279, 90, and 286 μs, respectively. **e** DEER measurements results signal of NV_A_–NV_C_, NV_C_–NV_B_, and NV_A_–NV_B_, when NV_A_, NV_C_, NV_A_ were used as sensor. Closed circles represent the DEER data and solid lines show the fitting curves. The *ν*_dip_ of these pairs were evaluated to be 53.0, 24.1, and 4.6 kHz, respectively. **f** The DEER measurement results signal of NV_C_–NV_A_, NV_B_–NV_C_, and NV_B_–NV_A_, when NV_C_, NV_B_, NV_B_ were used as the sensor, respectively. Similar *ν*_dip_ values of 56.6, 24.2, and 4.6 kHz were evaluated

While the triple NV centres were found as shown in Figs. 1c and 2a, three NV centres in optical spots do not mean coupled triple NV centres. The dipole–dipole interactions among three NV centres are required to realize a quantum register. In this paper, we denoted a triple as a dipole–dipole interaction that measured at least two of the three pairs as a coupled triple. As shown in Fig. 3a, the pairs in the coupled triple were divided into strongly coupled and weakly coupled. A strongly coupled triple was defined as at least two of the three pairs having strong coupling. Here, DEER measurements were used for evaluating the dipole–dipole interactions between NV–NV. As a result, seven of the nine triples were categorized as not coupled triple. The residual two triples were categorized as a weakly coupled triple or a strongly coupled triple. The former weakly coupled triple is presented in the Supplementary Information (Supplementary Figs. 3 and 4). The latter strongly coupled triple is discussed in the paper in greater detail.

The three NV centres in the triple NV centre were labelled as NV_A_, NV_B_, and NV_C_, as shown in Figs. 1c and 3b. The orientations of the three NV centres were different, and the resonant frequencies for NV_A_, NV_B_, and NV_C_ were evaluated to be 2750, 2795, and 2846 MHz, respectively. The three NV centres can be distinguished by their resonant frequencies, even if these centres are found in a fluorescence spot. In this study, DEER measurements were used to investigate the dipole coupling between two of the three NV centres. Figure 3c shows the pulse sequence for the DEER measurements. The dipole coupling strength, *ν*_dip_, between the two NV centres can be evaluated by using one NV centre as a sensor, NV_sensor_, and another NV centre as an emitter, NV_emitter_. The pulse pattern for NV_sensor_ was the same as that for the Hahn echo measurement, but the evolution time was fixed at *τ*_0_. The pulse pattern for NV_emitter_ was as follows: the electron spin of NV_emitter_ was initialized by a laser. After the subsequent evolution time, *τ*_1_, followed by a π-pulse of NV_sensor_, the emitter spin was flipped with a π-pulse of NV_emitter_. By varying *τ*_1_, the oscillation of the NV_sensor_ signal was observed if *ν*_dip_ was higher than the decoherence rate, defined as 1/*T*_2,Hahn_, of NV_sensor_. The oscillation frequency corresponds to *ν*_dip_ between NV_sensor_ and NV_emitter_.

Figure 3d and Supplementary Figure 5 show the Hahn echo decay curves observed from NV_A_, NV_B_, and NV_C_. The decay curves were fitted by exp(−2*τ/T*_2,Hahn_), where *τ* is the evolution time^46^. The closed circles represent the measured data and the solid lines show the fitting curves. The evaluated coherence times for NV_A_, NV_B_, and NV_C_ were 279, 90, and 286 µs, respectively. The detection limits of *ν*_dip_ by using NV_A_, NV_B_, and NV_C_ as sensors were evaluated to be 3.6, 11.1, and 3.5 kHz. In the DEER measurements_,_ the evolution time for the NV centre sensor was fixed at *τ*_0_, and set at 100, 100, and 150 μs for the *ν*_dip_ measurements of NV_A_–NV_C_, NV_C_–NV_B_, and NV_A_–NV_B_ pairs, respectively.

Figure 3e and Supplementary Figure 6 show the DEER measurement results. The closed circles represent the measured data and the solid lines show the fitting curves to cos(2π *τ*_1_ *ν*_dip_). *ν*_dip_, namely, 53.0 ± 0.1, 24.1 ± 0.3, and 4.6 ± 1.0 kHz, were observed for the NV_A_–NV_C_, NV_C_–NV_B_, and NV_A_–NV_B_ pairs when NV_A_, NV_C_, and NV_A_ were used as the sensor, respectively. The right side of Fig. 3f shows the DEER measurement results when the sensors and the emitters were flipped. *ν*_dip_, 56.6 ± 0.3, 24.2 ± 0.8, and 4.6 kHz, were observed for the NV_C_–NV_A_, NV_B_–NV_C_, and NV_B_–NV_A_ pairs, respectively. Similar *ν*_dip_ values were thus observed. Because the interrogation time of NV_B_–NV_A_ (sensor was NV_B_) was less than a half period of the modulation of the echo intensity, the fitting was not accurate, and the error could not be evaluated. The dipole coupling strength depends on the distance between the two spins, the angle between the two spin quantization axes, and the angle between each quantization axis and the connecting vector. The two spin quantization axes are determined by the crystal axes of the respective defects under the weak magnetic field condition. The dipolar coupling distance and the strength between the two spins show the maximum values when the two spins are axially aligned, which corresponds to a parallel configuration. The maximum distance between the coupled spins for a parallel configuration is estimated by the following equation^25^: $\nu _{{\mathrm{dip}}} = 2\frac{{\mu _0h\gamma ^2}}{{4\pi {\boldsymbol{r}}_{max}^3}}$, where *μ*_0_ = 4*π* × 10^−7^ H m^−1^ is the vacuum permeability, *h* *=* 6.626 × 10^−34^ J s is the Plank constant, *γ* *=* 28 GHz T^−1^ is the gyromagnetic ratio, and **r**_max_ is the maximum distance. The maximum distances when the dipolar coupling strength is 53.0, 24.1, and 4.6 kHz were evaluated to be 12.5, 16.3, and 28.2 nm, respectively. The assumption of a parallel configuration is inappropriate for the NV centre pair. The probability of forming a parallel configuration is extremely low because the orientation of the NV centre is fixed by a diamond lattice. Jakobi et al. calculated the median angular contribution when all four crystal axes and all directions of the **r** vector have equal probability at $\nu _{{\mathrm{dip}}} = 0.7\frac{{\mu _0h\gamma ^2}}{{4\pi {\boldsymbol{r}}_{median}^3}}$, where **r**_median_ is the median distance. The median distance of NV_A_–NV_C_, NV_C_–NV_B_, and NV_A_–NV_B_ were calculated to be 8.8, 11.5, and 19.9 nm, respectively.

## Discussion

One of the principle problems with the ion implantation technique is ion straggling, which results in inaccuracies in the targeting. The lower the ion energy, the smaller the straggling is. Low-energy ion implantation is widely used to create coupled NV centres and shallow NV centres.

**Fig. 4:**
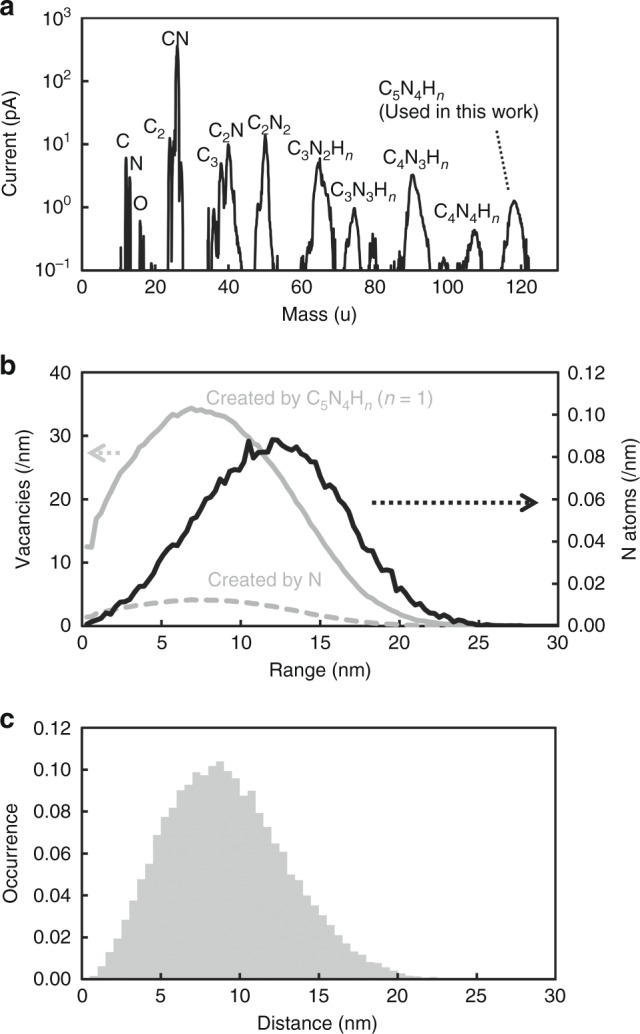
Features of the C_5_N_4_H_*n*_ ions from the adenine ion source. **a** Mass spectrum of ions from the adenine ion source. A C_5_N_4_H_*n*_ ion was used in this study. The mass of C_5_N_4_H_*n*_ was evaluated to be 118.2 ± 1.4 u. **b** Vacancy and nitrogen atom distributions calculated with the SRIM code. The dashed and solid grey lines show the distributions of vacancies created by the nitrogen and C_5_N_4_H_*n*=1_ ions. The solid black line shows the position of the implanted nitrogen atom. **c** The separation distance distribution between two nitrogen atoms when two nitrogen ions are implanted at the same point. The average distance and standard deviation of the implanted nitrogen atom were calculated to be 9 and 4 nm, respectively

A large number of nitrogen ions per nanohole were required for the coupled NV centre creation. For example, Jakobi et al. applied an implantation of the 30 keV ^14^N^+^ ion with 20 nitrogen ions through a nanohole with a diameter of 50 nm^25^. The distribution of nitrogen atoms in an implantation site is determined by both the straggling and the nanohole diameter. The fabrication of coupled triple NV centres and beyond by nanohole implantation has not yet been reported. One of the reasons for this is that the conversion ratio of implanted nitrogen to NV centres is low under the conditions needed to achieve shorter distances. The high dose implantation can fabricate triple NV centres and beyond, however, such intense beam implantation considerably shortens the coherence time. Therefore, methods for improving the creation yields and coherence time are needed. The efficient creation of coupled NV centre pairs was achieved via the implantation of nitrogen molecule ions with energies of 20 keV^33^. The distance between two implanted nitrogen atoms from a single molecule is determined by ion straggling. The separation of nitrogen atoms will be comparably minimized for molecular implantation compared to that of monoatomic ones through nanoholes. In short, a nitrogen molecule is regarded as an ideal point source. In terms of achieving short separation distances among NV centres, the implantation of cluster ions composed of only nitrogen atoms is more effective than the implantation via nanoholes; however, the difficulty of nitrogen cluster formation is a challenging issue. As an alternative to using ion species containing multiple nitrogen atoms, we used C_5_N_4_H_*n*_ ions from an adenine ion source instead of nitrogen cluster ions, as shown in Fig. 4a. Coupled NV centres were successfully created by C_5_N_4_H_*n*_ ion implantation and subsequent annealing.

The conversion efficiencies for the NV centres from implanted C_5_N_4_H_*n*_ ions were significantly higher than the previously reported values, as shown in Fig. 2b. The creation yields of single, double, and triple NV centres were 22.3%, 3.4%, and 0.1%, respectively. The total creation yield, which is the number of created NV centres divided by the number of implanted nitrogen ions (*n*_N_*N*_ion_), was 7.4%. Similar enhancement of the creation yield by the co-implantation of carbon ions, followed by nitrogen molecule ion implantation, has been reported^27,33,35^. The additional vacancies need to be provided sufficiently close to the implanted nitrogen. Otherwise, the increase in the yield due to co-implantation is mostly attributed to the capture of a vacancy by an ingrown nitrogen^47^. From the point of view of how vacancies are close to implanted nitrogen, the molecular ion implantation is much better than the standard co-implantation. Because the C_5_N_4_H_*n*_ ion is regarded as an ideal point source, the carbon atoms create vacancies as close to the implanted nitrogen as possible. A similar increase of the yield by supplying vacancies around the implanted nitrogen was reported for CN^−^ molecular ion implantation^27^. As shown in Fig. 4b, the vacancy density for C_5_N_4_H_*n*_ was seven times higher than that for single nitrogen ions. The co-implantation effect contributes considerably to the high creation yield. It was concluded that one of the most important features of C_5_N_4_H_*n*_ ion implantation is the high creation yield of NV centres. Furthermore, this high creation yield leads to the successful fabrication of double and triple NV centres.

The downside of the high creation efficiency resulting from dense vacancies is the presence of undesired defects, such as vacancy clusters [divacancy *V*_2_^0^ and 〈110〉 vacancy chains V_*n*_^0^ (*n* = 3, 4, ···, 7), *S* = 1], substitutional nitrogen (P1 centre, *S* = 1/2), or interstitial nitrogen with the 〈100〉 -nitrogen split interstitial structure (*S* = 1/2). We were concerned that NV centre decoherence would be caused by these undesired defects. First, the effects of substitutional and interstitial nitrogen atoms on *T*_2,Hahn_ were explored. Three, two, and one residual nitrogen atoms from the C_5_N_4_H_*n*_ ions would be located near single, double, and triple NV centres, respectively. If the residual nitrogen atoms disturbed the coherence of NV centres, the distributions of the *T*_2_,_Hahn_ histograms for single, double, and triple NV centres should show different trends. In this study, we assumed that the residual nitrogen atoms did not disturb the coherence of the NV centre, because the *T*_2,Hahn_ histograms for single, double, and triple NV centres showed similar trends, as shown in Fig. 2c. Second, the effect of vacancy clusters on *T*_2,Hahn_ was considered. The dense vacancies contribute to a high creation yield as discussed above but also created the potential for formation of vacancy clusters. Figure 2d shows that a reasonable coherence time was given compared with the previously reported data for random implantations of N^+^, N_2_^+^, and a nanohole implantation of N^+^. In short, there was no evidence that undesired defects, such as divacancies and vacancy clusters, remarkably disturbed the coherence properties of the NV centres. Surface spins are probably the main contributors to the decoherence of NV centres. Another investigation showed that noise spectroscopy based on the spectral decomposition of the echo decay of the dynamic decoupling measurement with the filter function of the pulse sequence can effectively separate the contribution of residual defects from those of surface spins^44^.

The coherence time and dipole coupling strength of triple NV centres, labelled NV_A_, NV_B_, and NV_C_, were investigated. We consider whether or not the triple NV centre can be used as quantum register. A quantum register consisting of *n*-qubits creates an *n*-qubit superposition state, including an entangled state, and allows for the implementation of a quantum algorism, such as quantum error correction. In a quantum register using coherent couplings of electron spins of multiple NV centres in a spin chain, a universal set of quantum gates are provided by single-qubit rotations and two-qubit gates, creating entanglement of the neighbouring two qubits, such as controlled-NOT (CNOT) gates. In an NV–NV pair, dipolar coupling, i.e., the conditional flip between two spins as observed in the DEER experiment, has been used for creating an entangled state of a two-qubit quantum register^7,8^. Strong coupling, *ν*_dip_ > 1/minimum(*T*_2A_, *T*_2B_), is suited for creating an entangled state. It should be noted that the coupling regime required to create an entanglement state is extended to weak coupling, *ν*_dip_ < 1/minimum(*T*_2A_, *T*_2B_), by using double quantum coherence^7^. Thus, the attainment of a strongly coupled condition, *ν*_dip_ > 1/minimum(*T*_2A_, *T*_2B_, *T*_2C_) for at least two NV–NV pairs in a triple NV centre brings us a step closer towards the realization of a three-qubit quantum register. The *ν*_dip_ of the triple NV centre were 53.0 kHz (NV_A_–NV_C_), 24.1 kHz (NV_C_–NV_B_), and 4.6 kHz (NV_A_–NV_B_), and 1/minimum (*T*_2A_, *T*_2B_, *T*_2C_) was 11.1 kHz. Therefore, the triple NV centre found here satisfied the condition required for three-qubit quantum register using coherent couplings of electron spins.

Further scaling-up of coupled NV centres by more than four would be partially unsolved. As shown in Fig. 2a, the creation yields of double and triple NV centres are one and two orders of magnitude lower than that of a single NV centre in the case of C_5_N_4_H_*n*_-65 keV implantation, respectively. The creation yield of four NV centres is expected to be one order of magnitude lower than that of triple NV centres, as it can be extrapolated from the creation yields of single, double, and triple NV centres. This issue is however potentially overcome, while the adenine complexes of dimer or trimers [C_5_N_5_H_5_]_*n*_, *n* ≥ 2 are practically achievable. Thus, it may be possible to fabricate an NV centre complex involving more than three neighbours with reasonable probability. In a single NV centre, the electron spin and the nuclear spins of nitrogen and proximal ^13^C nuclei comprise a hybrid quantum register^13,14^. Using such a hybrid register as a single node, the scaling up of the number of nodes by the dipolar coupling is an avenue to a larger register operated at room-temperature. The use of ^13^C-enriched adenine powder as the ion source could enable discrete ^13^C coupling to several NV centres. According to an SRIM simulation for 65 keV ^13^C_5_N_4_H_*n*_ ions, 3% of ^13^C atoms are located close to a nitrogen atom, with a distance of 3 nm.

## Methods

### C_5_N_4_H_*n*_ ion implantation

Adenine powder was used as the ion source. The ionized particles were electrically extracted and analyzed by mass spectrometry, as shown in Fig. 4a. A wide variety of ions, including nitrogen compound ions, were observed. In this study, 65 keV-C_5_N_4_H_*n*_ ions were used for implantation. The mass of the C_5_N_4_H_*n*_ ions was estimated to be 118.2 ± 1.4 u. The number of hydrogen ions, *n*, varied up to five. The beam current of the C_5_N_4_H_*n*_ ions was measured with a Faraday cup with an area of 0.64 cm^2^, and a beam current of 1 pA was observed. The beam flux was calculated to be 1 (pA)/0.64 (cm^2^)/1.6 × 10^−19^ (C) = 10^7^ cm^−2^ s^−1^. The fluences of 10^8^ and 10^10^ cm^−2^ were achieved by implantation times of 10 and 1,000 s, respectively. Usually, a uniform beam is achieved by the scanning beam at the expense of beam current. In this experiment, however, the ion beam was not scanned because the current of scanned beam was too small to be detected by the Faraday cup. According to the yield estimation by Poisson fitting as shown in Fig. 2a, the number of implanted C_5_N_4_H_*n*_ ions in 10,400 µm^2^ was estimated to be 7,116, which was 30% less than the number predicted from the beam fluence (10^8^ cm^−2^ × 10,400 µm^2^ = 10,400 ions).

The nitrogen and vacancy distributions were calculated with the SRIM code (version 2008)^38^, as shown in Fig. 4b. In this simulation, the energies of the nitrogen, carbon, and hydrogen were assumed to be 7.7, 6.6, and 0.6 keV, respectively. The vacancy density created by C_5_N_4_H_*n*=1_ was seven times that created by a single nitrogen ion. The number of vacancies created by a hydrogen ion was two orders of magnitude less than that created by a nitrogen ion, therefore the contribution of hydrogen numbers of up to five to the vacancy distribution created by C_5_N_4_H_*n*_ was negligibly small. The projected range and in-depth straggling of ^14^N-7.7 keV were calculated to be 11.4 and 9.3 nm, respectively. In addition to the straggling, the expected distribution and mean distance between the two nitrogen atoms were also calculated using SRIM code, because the distance between the stopped positions of the nitrogen atoms is important in this experiment. Figure 4c shows the simulated probability distribution of the distance between the two nitrogen atoms arising from the same molecule. The averaged value and standard deviation were calculated to be 9 and 4 nm, respectively.

### Sample preparation

The sample used in this study was a type IIa (100) single-crystalline diamond film that was homoepitaxially grown on a high-pressure high-temperature type Ib substrate via microwave-plasma-assisted CVD^48^. The CVD film thickness was 20 µm. To suppress the effects of ^13^C on the coherence properties of the NV centres, a ^12^C-enriched (99.95%) high-purity (nitrogen concentration <1 ppb) diamond was used.

After ion implantation, the sample was annealed at 1000 °C for 2 h in a forming gas (4% H_2_ in Ar) to create NV centres and recover the diamond lattice. We performed oxygen termination treatment and ozone exposure to improve the coherence properties of the NV centre^45,49^. The sample was annealed in an oxygen environment at 465 °C for 4 h, followed by cleaning in a 1:3 mixture of HNO_3_ and H_2_SO_4_ at 200 °C for 30 min.

### Room-temperature CFM

A laboratory-built CFM system was used to observe the NV centres. A series of 532-nm laser excitation and microwave pulses were used for initialization, coherent manipulations, and readout of the spin state. Excitation laser irradiation was performed via an air objective lens (×50, NA = 0.95), and luminescence was detected by an avalanche photodiode after passing through a pin-hole. The typical photon count from a single NV was 5 × 10^4^ c s^−1^. All optical experiments were performed at room-temperature with the application of a static magnetic field (~9 mT).

## Supplementary Information

Supplementary Information

Source Data

## Data Availability

The data that support the findings of this study are available from the corresponding author upon reasonable request.
