# Supplementary material for: Triple nitrogen-vacancy centre fabrication by C5N4Hn ion implantation
Source: Nat Commun. 2019 Jun 13;10:2664. doi: 10.1038/s41467-019-10529-x (PMC6565727; doi:10.1038/s41467-019-10529-x)
Supplement: Supplementary file 1 — Supplementary Information [file 41467_2019_10529_MOESM1_ESM.pdf]

SUPPLEMENTARY INFORMATION

Triple nitrogen-vacancy centre fabrication by $C_5N_4H_n$ ion implantation

Haruyama *et. al.*

Supplementary Figures

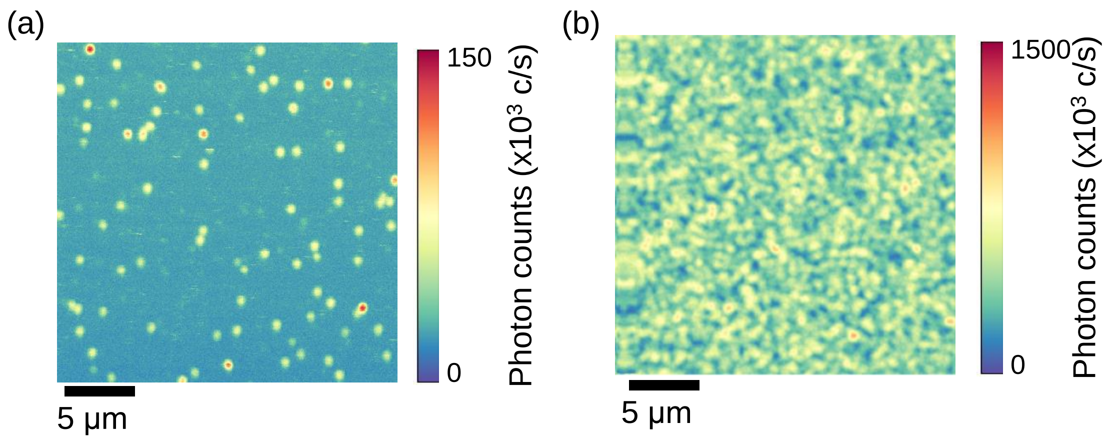

Supplementary Figure 1: Typical CFM images of $C_5N_4H_n$ implanted regions with a fluence of ~10^8 cm^{-2} (a) and ~10^{10} cm^{-2} (b).

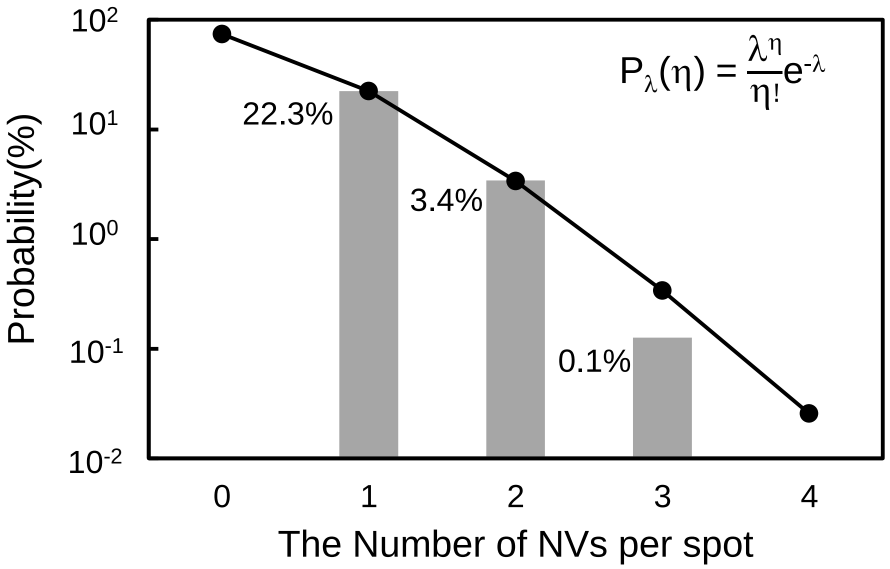

Supplementary Figure 2: The histogram of number of NV centres per spot. The measured number of NVs per spot for single, double, and triple NV were observed to be 1589, 244, and 9, respectively. The total number of NV centres in 10400 μm^2 was 2104. The number of NVs per spot followed Poisson distribution, which is expressed by following equation; $P_\lambda(\eta) = (\lambda^\eta/\eta!)\ exp(-\lambda)$, where $P_\lambda(\eta)$ is probability, λ is the average NVs per spot, η is the number of NV per spot, respectively. The measured number of NVs per spot were fitted with above equation by minimizing RSS (residual sum of squares). In the fitting, the average NVs per spot, λ, and the total number of incident molecules were left as free parameters. The closed circles and solid line show the best fitting Poisson distribution. As a result, the best fitting Poissonian had an average of 0.30 NVs/spot and the total number of 7116 molecules. The zero NV was created from 5274 implanted sites. The creation yield was evaluated to be 2104 NVs/(7116 molecules × 4 nitrogen in molecule) = 7.4 %.

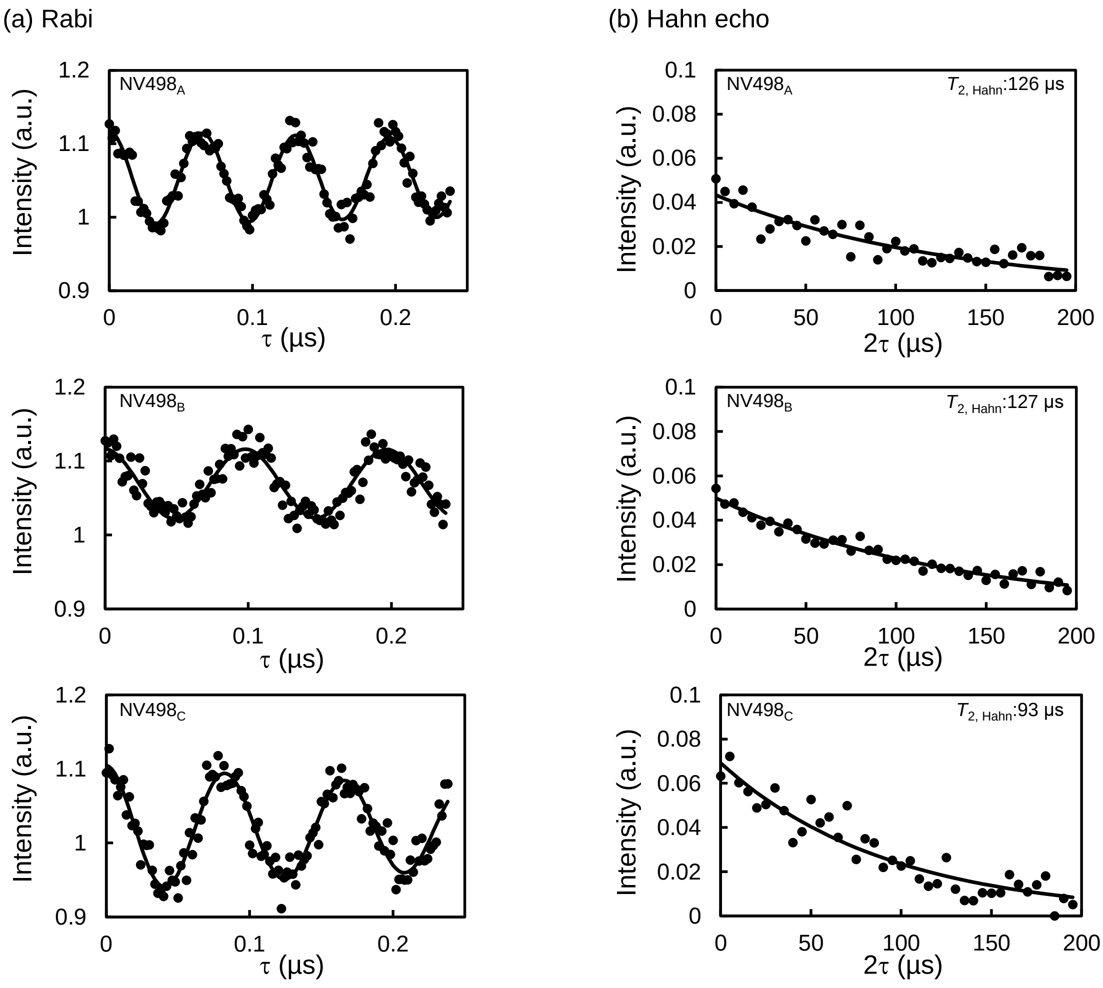

Supplementary Figure 3: Rabi oscillations and Hahn echo decays of NV498. NV498 contains three NV centres with the different axes. NV centres of axis i, ii, and iv were labelled as $NV498_A$, $NV498_B$ and $NV498_C$, respectively. (a) Rabi oscillations of $NV498_A$, $NV498_B$, and $NV498_C$. Closed circles represent the experimental data, and solid lines show fitting curves. The contrasts of Rabi oscillation were 11.1, 8.7, and 16.7 %, respectively. The pulse width of 2π were 65, 99, and 84 ns. (b) Hahn echo decays of $NV498_A$, $NV498_B$, and $NV498_C$. Closed circles represent experimental data and solid lines show exponential fitting curves. The values of $T_{2,Hahn}$ for $NV498_A$, $NV498_B$, and $NV498_C$ were evaluated to be 126.0 μs, 127.3 μs, and 92.9 μs, respectively.

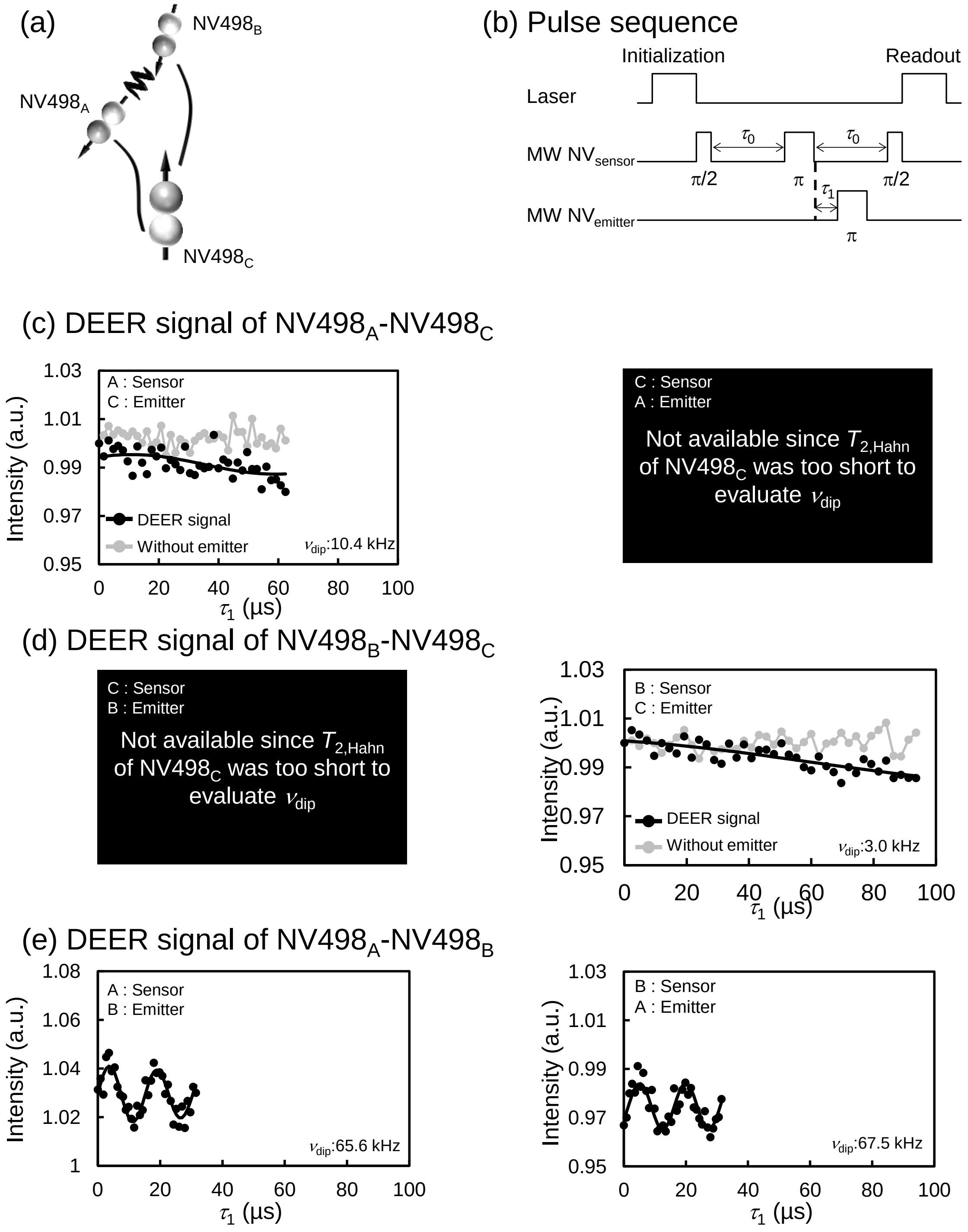

Supplementary Figure 4: Double electron-electron spin resonance (DEER) signals of NV498. (a) Schematics of triple NV (labelled as NV498). (b) Pulse sequence of DEER measurement. (c) DEER signal of $NV498_A$-$NV498_C$ when $NV498_A$ was used as sensor (left side). The black circles and line show DEER signal. The weak Echo modulation (ν_{dip} = 10.4 kHz) was observed for $NV498_A$ when the coupled $NV498_C$ was flipped within the echo sequence. A control experiment with a detuned driving field for $NV498_C$ was also shown by gray circles and line. In the detuned

case, $NV498_C$ was not flipped and thus the fluorescence intensity kept the constant values. (d) DEER signal of $NV498_B$-$NV498_C$ when $NV498_B$ was used as sensor (right side). The weak modulation (ν_{dip} = 3.0 kHz) was observed in the same manner as $NV498_A$-$NV498_C$. Because the coherence time of $NV498_C$ was too short to evaluate ν_{dip} of both $NV498_A$-$NV498_C$ and $NV498_B$-$NV498_C$, the any signal was not observed as shown in the right side of the figure (c), and the left side of the figure (d). (e) DEER signals of $NV498_A$-$NV498_B$ when $NV498_A$ was used as sensor (left side) and $NV498_B$ was used as sensor (right side). The closed circles show data and the solid lines show fitting curves. As shown, the clear modulations were observed and ν_{dip} were evaluated to be 65.6 kHz, and 67.5 kHz, respectively.

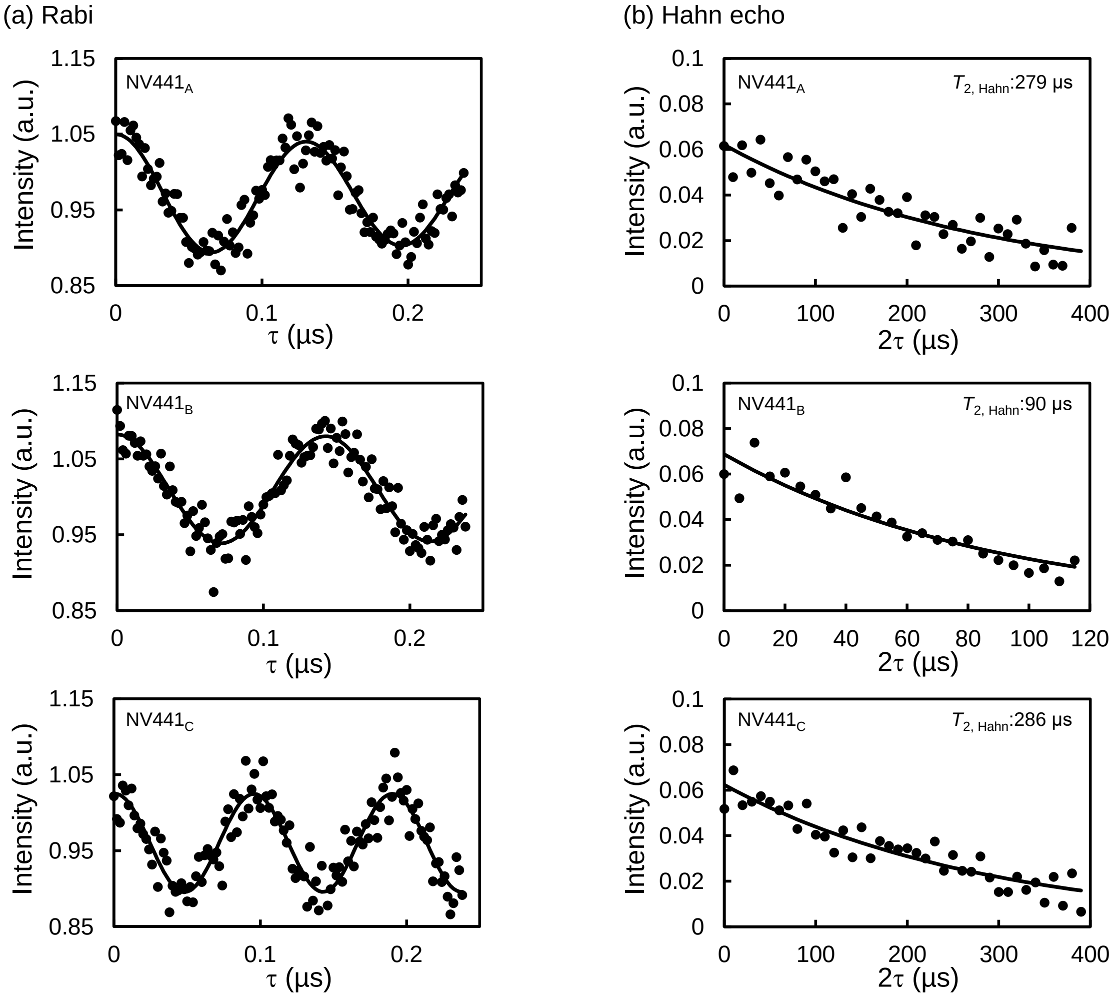

Supplementary Figure 5: Rabi oscillations and Hahn echo decays of NV441. NV441 contains three NV centres with the different axes. NV centres of axis ii, iii, and iv were labelled as $NV441_A$, $NV441_B$ and $NV441_C$, respectively. (a) Rabi oscillations of $NV441_A$, $NV441_B$, and $NV441_C$. Closed circles represent the experimental data, and solid lines show fitting curves. The contrasts of Rabi oscillation were 16.6, 13.9, and 13.4 %, respectively. The pulse width of 2π were 130, 142, and 95 ns. (b) Hahn echo decays of $NV441_A$, $NV441_B$, and $NV441_C$. Closed circles represent experimental data and solid lines show exponential fitting curves. The values of $T_{2,Hahn}$ for $NV441_A$, $NV441_B$, and $NV441_C$ were evaluated to be 278.6 μs, 90.4 μs, and 285.5 μs, respectively.

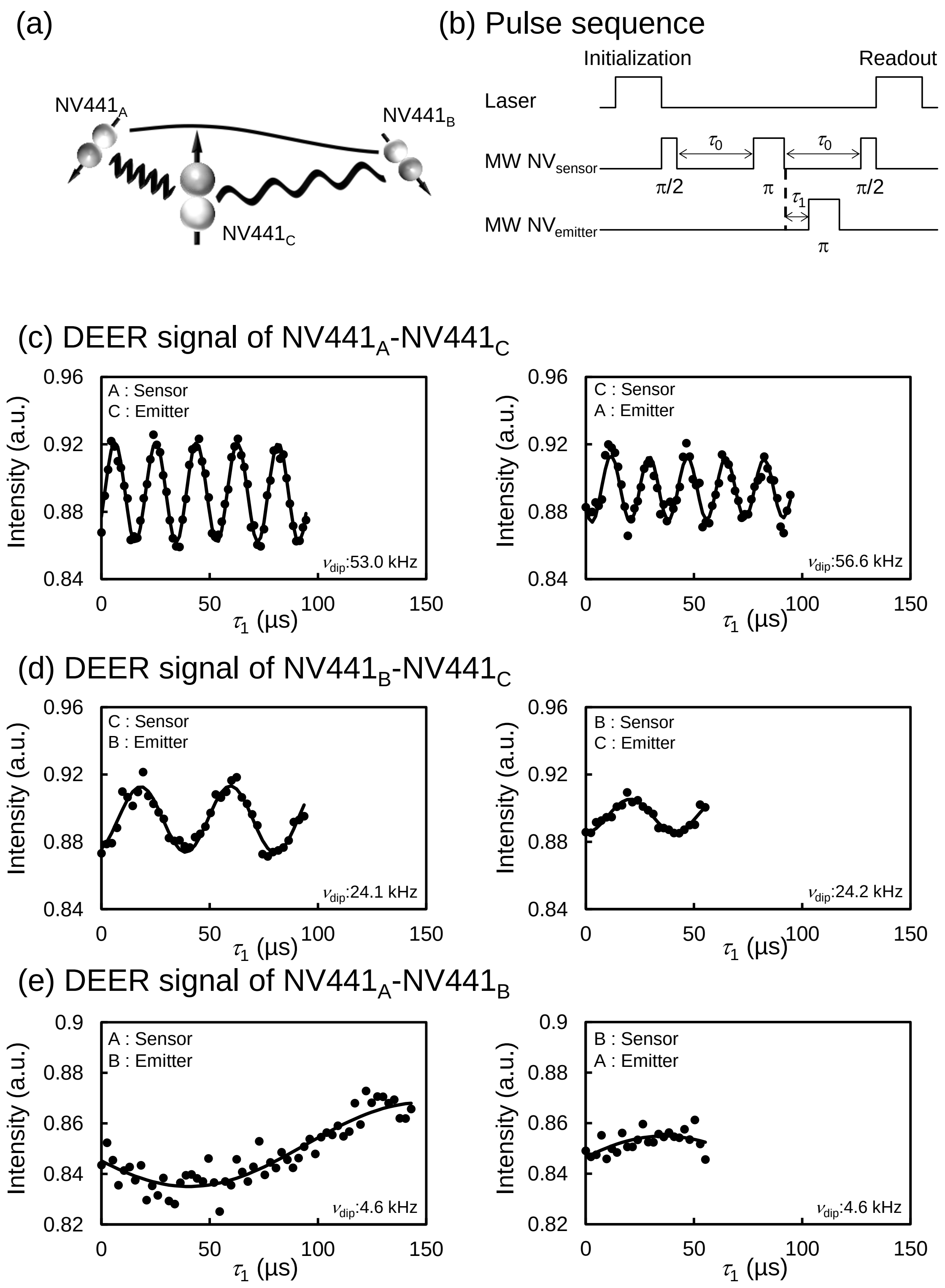

Supplementary Figure 6: DEER signals of NV498. (a) Schematics of triple NV (labelled as NV441). (b) Pulse sequence of DEER measurement. (c) DEER signals of NV441$_A$-NV441$_C$ when NV441$_A$ was used as sensor (left side) and NV441$_C$ was used as sensor (right side). The closed

circles show data and the solid lines show fitting curves. The clear modulations were observed and ν_{dip} were evaluated to be 53.0 kHz, and 56.6 kHz, respectively. (d) DEER signals of $NV441_B$-$NV441_C$ when $NV441_C$ was used as sensor (left side) and $NV441_B$ was used as sensor (right side). The closed circles show data and the solid lines show fitting curves. The clear modulations were observed and ν_{dip} were evaluated to be 24.1 kHz and 24.2 kHz, respectively. (e) DEER signals of $NV441_A$-$NV441_B$ when $NV441_A$ was used as sensor (left side) and $NV441_B$ was used as sensor (right side). The closed circles show data and the solid line shows fitting curves. As shown, the weak modulations were observed and ν_{dip} were evaluated to be 4.6 kHz and 4.6 kHz, respectively.

Supplementary Table

Supplementary Table 1: List of coherence times measured by Hahn echo pulse sequence. "NA" means that $T_{2,\,\mathrm{Hahn}}$ was not evaluated because of unstable charge state of NV centre. The left column of label shows the number of NV centres in an optical spot. Nine triples were found, and seven of nine triples were categorized as "not coupled". The residual two triples were categorized as "weakly coupled" or "strongly coupled".

Label		Axis			
		i	ii	iii	iv
One	NV83	208.6			
	NV689	117.8			
	NV690	2.4			
	NV691	15.3			
	NV692	112.7			
	NV693	48.3			
	NV694	2.6			
	NV695	49.0			
	NV696	146.2			
	NV697	28.9			
	NV698	120.8			
	NV699	23.9			
	NV700		85.2		
	NV701		15.6		
	NV702		104.8		
	NV703		143.0		
	NV704		56.3		
	NV705		74.2		
	NV706		11.4		
	NV707		42.2		
	NV708		56.9		
	NV709		31.6		
	NV710		6.8		
	NV711				2.3
	NV712				103.6
	NV713				13.7
	NV714				2.1
	NV715				4.0
	NV716				22.3
	NV717				255.4
	NV718				165.7
	NV719				183.1
	NV720				89.6
	NV721			1.8	
	NV722			2.8	
	NV723			428.0	
	NV724			241.0	
	NV725			0.5	

Label		Axis			
		i	ii	iii	iv
Two	NV470	25.3	11.4		
	NV476	1.8	8.2		
	NV483	166.8	13.4		
	NV486		48.3		91.5
	NV490	12.2			2.4
	NV496			38.0	257.9
	NV499	103.6			173.4
	NV500	35.1			217.0
	NV507			13.6	5.9
	NV510			86.2	65.8
	NV511		221.3	44.8	
	NV513			3.4	5.0
	NV726		38.8		36.2

Label		Axis			
		i	ii	iii	iv
Three	NV22	NA		207.7	NA
	NV335	NA	322.8	33.7	
	NV343	2.0	NA	20.9	
	NV371	411.5		296.2	NA
	NV397	NA	23.9		168.7
	NV441		278.6	90.4	285.5
	NV462	NA	142.6	194.1	
	NV463	43.0	NA	37.3	
	NV498	126.0	127.3		92.9
